# Individualized surgery combined with radiotherapy and triamcinolone acetonide injection for the treatment of auricular keloids

**DOI:** 10.1186/s12893-021-01253-9

**Published:** 2021-05-22

**Authors:** Qiang Sun, Er-te Yu, You Zhou, Shuang Tong, Kai-jian Zhou, Shu Guo

**Affiliations:** Department of Plastic Surgery, The First Hospital of China Medical University, 155 Nanjing North Road, Heping District, Shenyang, 110001 China

**Keywords:** Auricular keloid, Individualized surgery, Radiotherapy, Triamcinolone acetonide

## Abstract

**Background:**

Although multiple methods have been proposed to treat auricular keloids, low curative effects and high recurrence rates are currently major clinical problems. Thereinto, surgery combined with radiotherapy and triamcinolone acetonide injection is considered to be the proper choice for comprehensive treatment of auricular keloids. This study aimed at evaluating the therapeutic effect of individualized surgery combined with radiotherapy for the treatment of auricular keloids.

**Methods:**

From February 2014 to February 2017, 67 patients with 113 auricular keloids in total were enrolled in this study. According to specific conditions of lesions, the local tissue and patients’ individual wishes, different surgical methods were selected to analyze the scar excision and repairment of the defect. Within 24 h after the keloid was excised, 5 MeV electron beam irradiation by the linear accelerator was used by radiotherapy with a total dose of 20 Gy at interval of 1 day for 10 consecutive times. Triamcinolone acetonide was injected immediately after surgery, and per month afterward in the following three months.

**Results:**

113 keloids in total were received treatment. The follow-up period was 24 months. Fourteen keloids (12.39%) showed subjective recurrence with a success rate of 87.61%. Wilcoxon matched-pairs rank-sum test was used to compare the differences of the 24-month postoperative VSS scores and the preoperative VSS scores. The VSS scores were as follows: 82 keloids (72.57%) scored less than 5 points (good result), 21 keloids (18.58%) scored 6 to 10 points (fair result), and only 10 keloids (8.85%) scored more than 10 points (bad result). The effective rate was 91.15%.

**Conclusions:**

Individualized surgery combined with early postoperative radiotherapy and triamcinolone acetonide injection is an ideal treatment method to ensure good auricular appearance, low incidences of complications and recurrence based on effective treatment of auricular keloids.

## Background

Skin scars are the inevitable results of tissue repair after a certain degree of trauma. Keloid occurs when skin fibers abnormally proliferated and the wounds were repaired excessively [1]. Its appearance is unsightly that it causes discomfort to patients with various degrees of dysfunction. If it ruptures, it tends to become an unhealed ulcer and may even become malignant [1, 2]. Auricular keloids are usually caused by ear piercing, infection, surgery and trauma, and so on, with high risks of local malformation, complications and recurrence. In addition, it often proliferates and enlarges for many years, or even invades the surrounding normal skin, seriously affecting the appearance [3].

Effective treatment relies to a large extent on the understanding of pathogenesis, but the pathogenesis is so complex that it has not yet been fully revealed. As a result, the treatment is greatly restricted. At present, there are many means of treatments for auricular keloids, but treatment effects were poor and recurrence rates were high [3, 4]. It was reported that the recurrence rate of surgery alone was as high as 45–100%, and that of glucocorticoid injection alone in lesions was 9–50% [3, 5]. Therefore, most scholars currently advocated comprehensive treatment [6–8], which will conducted surgery first, and then combined with some relevant auxiliary treatments, such as hormones, pressure therapy, radiotherapy, and so on. Among them, surgery combined with radiotherapy and triamcinolone acetonide injection might be the preferred scheme of comprehensive treatment for auricular keloids [7–9].

## Patients and methods

### Patient group

Patients were selected from the Department of Plastic Surgery, the First Hospital of China Medical University between February 2014 to February 2017. The study was approved by ethical review board of China Medical University, which followed the ethical principles of the Declaration of Helsinki 1964, and all the patients provided the written informed consent.

### Inclusion criteria

All the patients were diagnosed with auricular keloids:

1. The scar proliferation lasted for more than 12 months after formation, and there was no sign of stop.
2. The scar was flush and higher than the surface of the normal skin, proliferating like a tumor to attract the surrounding normal skin and extend beyond the border of the original wound, causing itching and pain.
3. Recurrence of patients after surgical excision, freezing, laser, hormone or radiotherapy was also included.

### Treatment protocol

#### Surgical Therapy

**Fig. 1:**
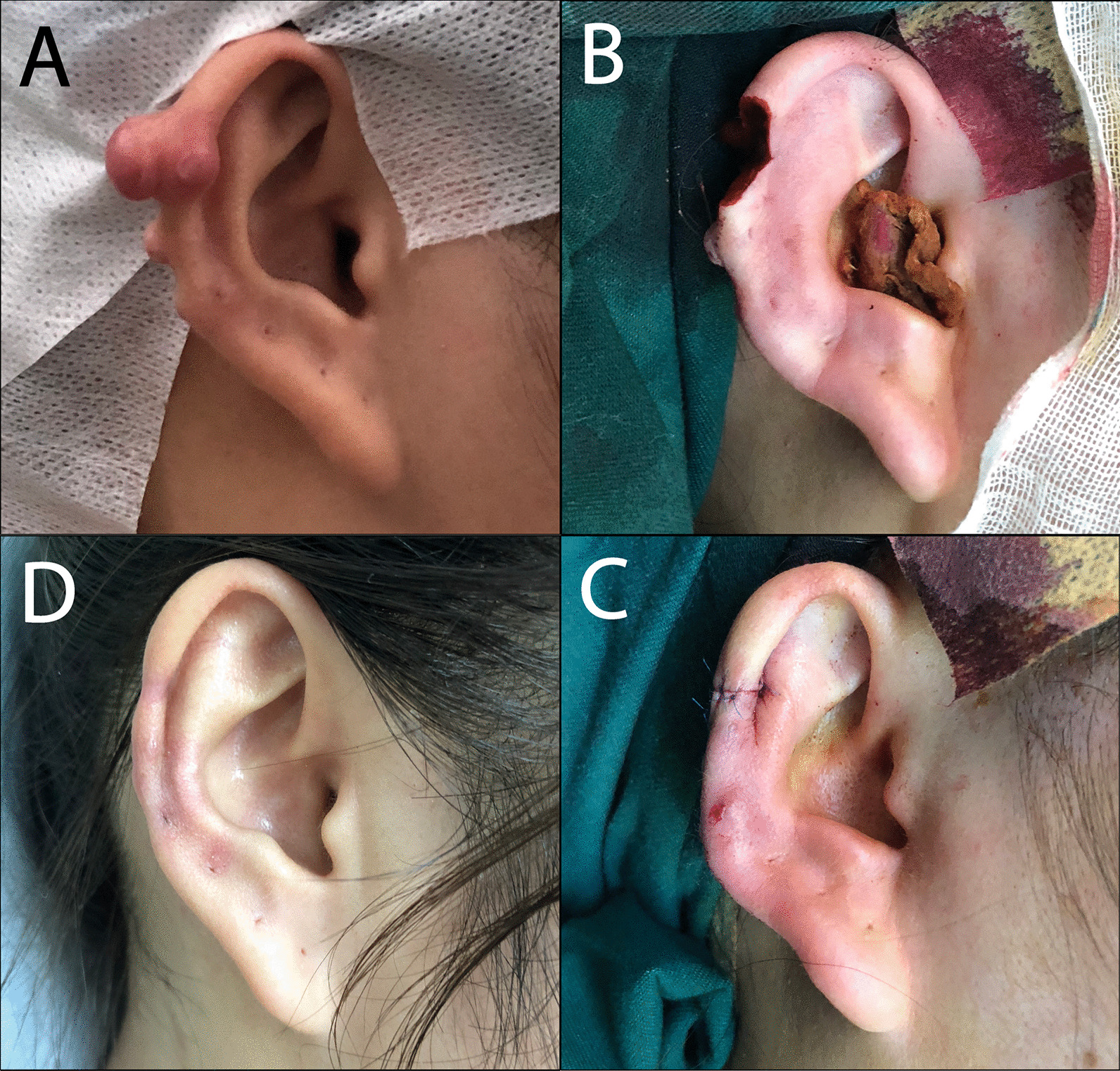
This female was underwent immediate suture after excision (I). **A** preoperation; **B** keloid and part auricular cartilage were resected together; **C** immediately after operation; **D** 12 months after operation

**Fig. 2:**
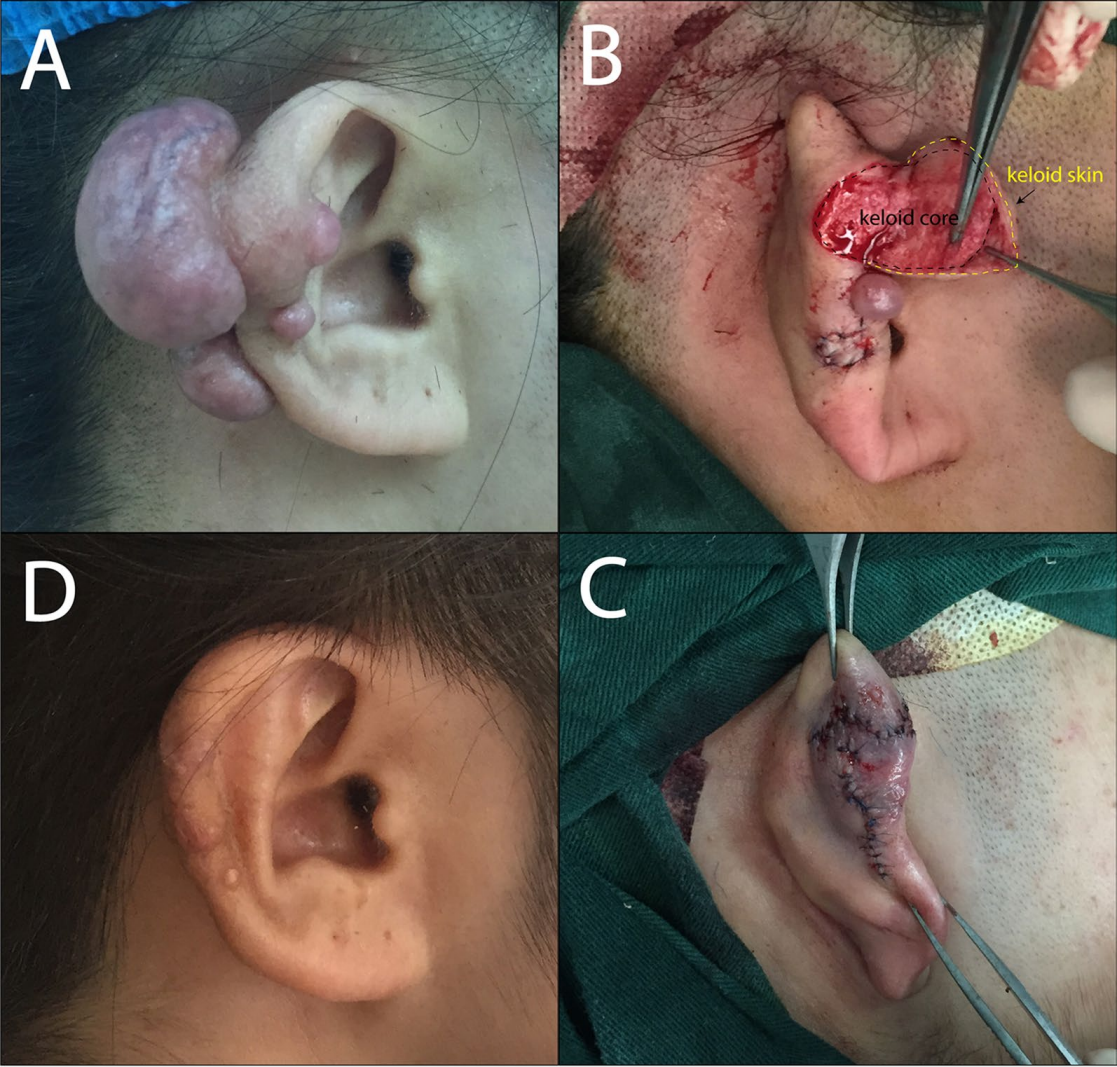
This female was underwent keloid core excision and scar flap plasty (II). **A** preoperation; **B** designed "C" shape incision; **C** immediately after operation; **D** 12 months after operation

**Fig. 3:**
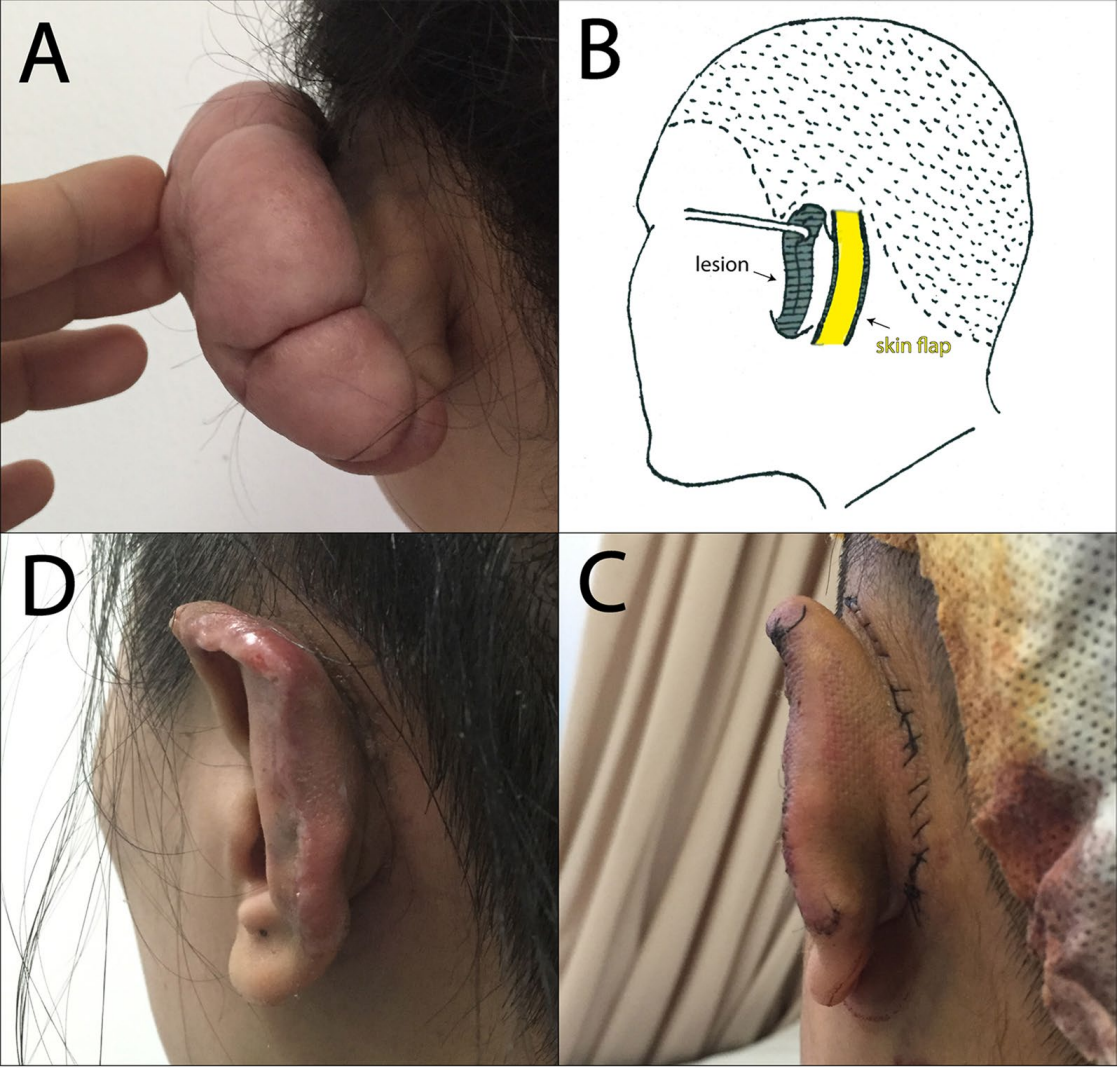
This female was underwent complete keloid excision and adjacentflap plasty (III). **A** preoperation; **B** designed postauricular flap with two pedicles; **C** immediately after operation; **D** 12 months after operation

1. Suture immediately after excision (I)) For the small basal area of keloid, if the suture immediately after excision could have very little effect on the appearance of auricle, we could completely resect the keloid along the outer edge of the keloid or directly performed the fusiform excision. Sometimes, part of auricular cartilage needed to be resected together (Fig. 1).
2. Keloid core excision and scar flap plasty (II): For large basal area of keloid, if the suture immediately after excision had a great influence on the appearance of auricle, we could perform keloid core excision and scar flap plasty. We designed a "C" shape incision along the trailing edge of keloid or fusiform incision on keloid surface design. We then performed a sharp dissection between the keloid skin and the keloid core, and between the keloid core and the ear cartilage, respectively. The keloid core excision and the scar flap formation were completed at the time. Finally, we covered the ear cartilage with a scar flap (Fig. 2).
3. Complete keloid excision and adjacent flap plasty (III): For large basal area of keloid, if the suture immediately after excision could have a great effect on the appearance of auricle, but the patient urges complete keloid excision, we could excise keloid completely along keloid outer edge, and then use local advancing flap or rotating flap, such as postauricular flap, to repair ear deformation (Fig. 3).
4. Among all operations, 1% lidocaine was used for local infiltration anesthesia, and a 6–0 nylon thread was applied to the skin. In addition, a local compression bandage was used appropriately, and stitches was taken 7 days after the operation.

#### Radiotherapy

All patients received 5 MeV electron beam irradiation by the linear accelerator (Artiste, Siemens, Germany) within 24 h after excision of the keloid. According to the conventional standard of the department of radiotherapy in our hospital, the total dose was 20 Gy (2 Gy for each time), and times of irradiation was 10 (1 day interval) in total. The margin of the irradiated area was 0.5–1 cm around the scar, and all wounds were radiated when a local flap was used.

#### Corticosteroid injection

In this study, 1 mL of triamcinolone acetonide injection (Kunming Jida Pharmaceutical Co., Ltd., National Pharmaceutical Standard H53021604, 40 mg/mL) and 1 mL of 2% lidocaine injection (Shanghai Zhaohui Pharmaceutical Co., Ltd. National Pharmaceutical Standard H31021072, 100 mg/5 mL) were mixed with a 2.5 mL syringe to prepare for a 1:1 ratio mixture. The mixed solution in a 1 mL syringe was injected in parallel to the edge of the scar and the surrounding skin immediately after surgery, and per month in the following three months after the surgery. The drug was uniformly and slowly injected into the scar at a distance of 1 cm, and 0.1 mL of the mixture was injected at each point. The infiltration range was 0.5 cm in diameter until the lesion became white.

### Therapeutic effect evaluation standard and statistical analysis

According to Masoodi Z [10], subjective recurrence was diagnosed when the lesion surfaced at the same site, which was difficult to feel during basic clinical examination, but it was similar to the previous lesion in patient’s perspective, and the lesion remained at 24 months of follow-up.

The lesion was further measured in the following 24 months using the Vancouver Scar Scale (VSS). Scores were tallied by different observers. The observers did not know pretreatment scores, which were categorized as follows: 0–5: Good Outcome; 6–10: Fair Outcome; and > 10: Poor Outcome. Effective rate = (Good Outcome + Fair Outcome)/ Poor Outcome. Herein, 24 month postoperative VSS score was compared to the preoperative VSS scores.

All data were entered into a database and processed by the Statistical Package Social Sciences (SPSS version 18.0; SPSS Inc, Chicago). Wilcoxon matched-pairs rank-sum test was used to compare the differences between the 24-month postoperative VSS scores and the preoperative VSS scores. *P* < 0.05 was considered as statistically significant.

## Results

67 cases of auricular keloid patients (11 males and 56 women) with 113 auricular keloids were treated in this study, including 63 keloids in left ear, 50 in right ears, 65 in helix, and 45 in earlobe. The size was between 0.2 × 0.2 × 0.2 cm and 4.0 × 4.0 × 2.0 cm. Patients ranged in age from 18 to 50 years old, with an average age of 28.0 years old. The primary cause of auricular keloids were 60 cases of ear piercing, 5 cases of trauma, and 2 cases of surgery. The medical history ranged from 1 to 18 years. Before our treatment, 10 patients underwent surgical excision, 2 patients received external drug treatment, 3 patients received hormone treatment, and 2 patients received laser treatment, but all these treatments were ineffective.

According to the above treatments, 52 cases of keloids were sutured immediately after excision (I), 43 cases of keloids were treated with core excision and scar flap plasty (II), 18 cases of keloids were completely treated with keloid excision and adjacent flap plasty (III). The typical cases were shown in Fig. 1, 2 and 3. All patients were cured well without any complication. Radiotherapy was treated within 24 h after surgery, and triamcinolone acetonide was injected immediately after surgery, and per month after the following three months after surgery. The follow-up period was 24 months.

Subjective recurrence occurred in 14 cases of keloids (12.39%). Among them, 6 of 52 keloids (11.54%) sutured immediately after excision (I), 6 of 43 keloids (13.95%) underwent keloid core excision and scar flap plasty (II), and 2 of 18 keloids (11.11%) underwent complete keloid excision and adjacent flap plasty (III).

The VSS scores showed that 82 cases of keloids (72.57%) scored less than 5 points (good outcome), 21 cases of keloids (18.58%) scored 6 to 10 points (fair outcome), and only 10 cases of keloids (8.85%) scored more than 10 (bad outcome). The effective rate was 91.15% (Table 1).

**Table 1:** Therapeutic effect evaluation by VSS

Therapeutic effect (*n*, %)				Scores				
Good	Fair	Poor	Effective rate	Mean of Preoperative VSS	Mean of Postoperative VSS (at 2 y)	Z(Wilcoxon Matched-Pairs rank-sum test)	*P*	
82 (72.57)	21 (18.58)	10 (8.85)	91.15	10.81	4.02	− 9.068	< 0.001	

## Discussion

Ears are the exposed part and the fine anatomic structures of the body. Fortunately, two ears are located on either side of the head, and a slight change will not be so obvious. Surgery is the most important part of the treatment of auricular keloids. Compared with some scholars who concreted numerical range and location of lesion compared to specific surgery [11, 12], we demonstrated specific surgical methods according to specific conditions, because no special operation could be considered as the best approach. For repairing the auricular defect, the available auricular tissue for surgery was extremely limited. Especially for large auricular keloids, complete excision might affect auricular appearance, and might cause protruding ears, cup ear, narrow cranio-auricular angle, bilateral obvious asymmetry deformity, and so on. Some scholars performed ear full-thickness wedge excision in the treatment of large auricular keloids as we did [13]. Although this method was performed very thoroughly, it was difficult to avoid damage to normal ear tissue. Even it might cause obvious asymmetry on both sides. Thus, we used this method for the small basal area of keloid.

Meanwhile, as we know, greater tissue tension would increase the risk of recurrence. Therefore, we retained tissue to the greatest extent and covered the wound with available tissue to maintain good auricular appearance without secondary deformities. It had been reported that the residual thin layer keloid tissue under scar flap did not increase the risk of recurrence [14–16]. For large basal area of keloid, we recommend the use of keloid core excision and scar flap plasty (II), which could ensure effective treatment of disease and good auricular appearance. The "C" shape incision was designed along the keloid trailing edge. Different to "X" shape incision, the purpose of “C” shape incision was that surgical scars might be located behind the ear [17]. Besides, the "C" shape incision was less than half of the keloid circumference to ensure the blood supplyment of scar flap. In particular, injection between the skin and keloid core could easily excise the keloid core. After the keloid core excision was completed, the excess scar flap tissues were resected and sutured without tissue tension, ensuring effective treatment of disease and good auricular appearance.

There is still a controversy about whether using radiotherapy may lead to malignant tumors. However, data suggested that there was no need to worry about whether it should be used indiscriminately [18, 19]. The best time of radiotherapy for auricular keloid in the literature is different. It was found that early postoperative radiotherapy could better prevent the recurrence [20]. Infantile fibroblasts appeared within 24 h after post-traumatic incision. If radiotherapy was used at this time, it could effectively inhibit the proliferation of fibroblasts and capillaries. Therefore, we should chose to start radiotherapy within 24 h after operation. Radiotherapy, triamcinolone acetonide injection should be also performed as early as possible. Therefore, in our study, triamcinolone acetonide was injected for the first time immediately after suturing.

113 keloids in total were received treatment. The subjective recurrence rate was 12.39%,while the success rate was 87.61%. Meanwhile, the objective recurrence rate (VVS scores) was 8.85%, while the effective rate was 91.15%. Compared with other reports, the recurrence rate after combined surgical excision and postoperative radiation varied from 2.8% to 71% [9, 11, 12], and the recurrence rates of our method were satisfied. However, the major limitation of our study is missing control group.

## Conclusions

Personalized medicine should be performed for auricular keloids, which not only considered the size of the lesions, but also considered patients’ wishes and the specificity of skins of different patients, such as tissue quantity, elasticity, tension, etc., which made the surgery more flexible and fully reflected the individual characteristics of patients during surgery. Furthermore, early postoperative radiotherapy and triamcinolone acetonide injection should be performed.

## Data Availability

The datasets used and analyzed during the current study are available from the corresponding author on reasonable request.
